# Clinical Characteristics, ERCP Management, and Early Adverse Events Among Patients with Choledocholithiasis at a Romanian Tertiary Referral Center: A Retrospective Cohort

**DOI:** 10.3390/diseases14090340

**Published:** 2026-09-16

**Authors:** Cristina Angelescu, Ioana Anca Bădărău, Radu Bogdan Mateescu

**Affiliations:** 1 Faculty of Medicine, “Carol Davila” University of Medicine and Pharmacy, 050474 Bucharest, Romania; anca.badarau@umfcd.ro (I.A.B.); bogmateescu@gmail.com (R.B.M.); 2 Department of Gastroenterology and Hepatology, Colentina Clinical Hospital, 020125 Bucharest, Romania

**Keywords:** choledocholithiasis, ERCP, acute cholangitis, tertiary referral cohort, biliary obstruction, post-ERCP acute pancreatitis

## Abstract

Background: Choledocholithiasis is a common cause of biliary obstruction and may lead to acute cholangitis, pancreatitis, and other serious complications. Despite the widespread use of endoscopic retrograde cholangiopancreatography (ERCP), data describing the clinical profile and management of patients with choledocholithiasis in Romania remain limited. Objectives: This study aimed to describe demographic characteristics, diagnostic work-up, procedural characteristics of ERCP, microbiological findings, and early clinical outcomes of patients with choledocholithiasis undergoing ERCP at a high-volume tertiary referral center. Methods: We conducted a retrospective, observational, single-center cohort study of adults with confirmed choledocholithiasis who underwent ERCP between January 2021 and September 2025. During the study period, 2514 ERCP procedures were performed at our center for all indications, of which 631 unique patients with choledocholithiasis fulfilled the eligibility criteria and were included in the final cohort. Demographic characteristics, comorbidities, clinical presentation, laboratory findings, imaging results, ERCP-related variables, and early procedural outcomes were analyzed descriptively. Results: The cohort included 631 patients with a median age of 68 years (IQR 56–77), and 59.9% were women. Abdominal pain (59.3%) and jaundice (48.2%) were the most common presenting symptoms. Acute cholangitis was present in 57.7% of patients; among these patients, severity according to the Tokyo Guidelines 2018 was mild in 52.7%, moderate in 10.7%, and severe in 36.6%. Computed tomography was the most frequent imaging modality confirming the diagnosis (42.5%), followed by magnetic resonance cholangiopancreatography (30.7%) and ultrasonography (26.8%). Median common bile duct diameter was 14 mm and median stone diameter was 7.9 mm; multiple stones or sludge were present in 56.7%. Difficult cannulation occurred in 10.8% of patients, mechanical lithotripsy in 19.5%, biliary stent placement in 18.9%, and post-ERCP pancreatitis in 10.8%. Intraprocedural bleeding occurred in 4.9% of patients, while post-ERCP bleeding occurred in 1.9% and no perforations were recorded. In-hospital mortality occurred in 2 patients (0.3%). Bile cultures were obtained in 52 patients (8.3%), of which 37 (71.2%) yielded bacterial growth, with *Escherichia coli* being the most frequently isolated microorganism. Conclusions: This study provides real-world evidence on the epidemiology, diagnostic evaluation, ERCP management, and early outcomes of choledocholithiasis in a Romanian tertiary referral center.

## 1. Introduction

Gallstone disease is among the most common digestive disorders, and the migration or formation of calculi in the common bile duct (CBD) causes choledocholithiasis. The condition is clinically consequential because biliary obstruction may range from self-limited biliary pain to obstructive jaundice, acute cholangitis, and acute biliary pancreatitis [1,2]. Timely diagnosis and appropriate selection for biliary drainage or stone extraction are therefore central to reducing morbidity and organizing care in high-volume referral settings.

The most comprehensive body of recent evidence comes from a 2024 systematic review and meta-analysis, which estimated the pooled global prevalence of gallstone disease at 6.1% [3]. This provides the epidemiological basis for choledocholithiasis: approximately 10–20% of patients with cholelithiasis have concomitant CBD stones, making the overall burden of choledocholithiasis substantial but difficult to quantify independently, as most population-based epidemiological data capture gallstone disease as a whole rather than CBD stones specifically [4]. The epidemiology of endoscopic retrograde cholangiopancreatography (ERCP) use for the treatment of choledocholithiasis has also changed substantially. Between 1998 and 2012, the proportion of hospitalized patients with cholangitis secondary to choledocholithiasis who underwent ERCP increased from 66.5% to 80.3%, with a greater proportion of procedures being performed emergently or within the first 48 h of hospitalization following the adoption of the Tokyo Guidelines criteria for acute cholangitis. This shift was associated with a reduction in mortality but also with increased healthcare costs [5].

The presentation of choledocholithiasis is heterogeneous. Some patients have biliary colic or transient biochemical abnormalities, whereas others present with jaundice, infection, or pancreatitis. This variation means that no single test is sufficient for every clinical situation and that the diagnostic pathway should reflect the patient’s pre-test probability of ductal stones. The European Society of Gastrointestinal Endoscopy (ESGE) recommends liver biochemical testing and abdominal ultrasonography as the initial evaluation for suspected common bile duct stones. When clinical suspicion remains despite inconclusive ultrasonography, endoscopic ultrasound or magnetic resonance cholangiopancreatography is recommended for confirmation [6]. This approach aims to identify patients who need therapeutic intervention while avoiding the risk of diagnostic ERCP in patients without a sufficiently likely or confirmed obstruction. Clinical, biochemical, and imaging variables are therefore combined in probability-based algorithms because no individual parameter is completely accurate [7,8].

ERCP now has a predominantly therapeutic role in confirmed or highly probable choledocholithiasis [9]. Endoscopic stone extraction can decompress the biliary tree and resolve obstruction through a minimally invasive approach. ESGE recommends offering extraction to patients with common bile duct stones who are fit to undergo intervention, including those without symptoms [6]. In acute cholangitis, the priority is biliary drainage, preferably by endoscopy. ESGE recommends drainage as soon as possible and within 12 h for patients with septic shock, within 48–72 h in moderate cholangitis, and electively in mild disease [6]. The urgency of ERCP thus depends not only on the presence of a stone, but also on the severity of infection and the patient’s physiological status.

ERCP management is not uniform across patients. The technical approach depends on stone size, number, and position; papillary and biliary anatomy; previous biliary interventions; and the patient’s comorbidities. Large, multiple, or impacted stones and altered anatomy can make duct clearance more difficult [10]. Given their referral role, tertiary centers are more likely to treat patients with complex choledocholithiasis, because patients may be transferred after failed initial treatment, with severe infection, or with difficult stone characteristics. ERCP is one of several management options, alongside laparoscopic CBD exploration and intraoperative ERCP, and the appropriate pathway depends on available surgical and endoscopic expertise [11].

Although ERCP provides substantial therapeutic benefit, it can cause clinically important early adverse events. Post-ERCP pancreatitis (PEP), bleeding, cholangitis, cholecystitis, and perforation are the principal complications. A recent systematic review and meta-analysis including 380 studies estimated PEP at 4.6% among all patients and 6.5% among patients undergoing their first ERCP. The pooled incidences of bleeding, cholangitis, and perforation were 1.5%, 2.5%, and 0.5%, respectively, while ERCP-attributable mortality was 0.2% [12]. The substantial variation across studies means that these values should be treated as population-level benchmarks rather than as rates directly transferable to a single institution. Case selection, indication, cannulation difficulty, procedural interventions, team experience, and the definitions and surveillance methods used for adverse events can all affect observed outcomes.

Early post-ERCP complications are influenced by patient, procedural techniques, and center-level factors. For PEP, studies identified younger age, prior acute pancreatitis or PEP, female sex, and sphincter of Oddi dysfunction as patient-related predictors of higher risk. The procedure-related factors supported by the most consistent evidence included guidewire passage into the pancreatic duct and first ERCP in a native papilla [13]. Other procedural features associated with an increased risk of PEP include main pancreatic duct (MPD) cannulation, pancreatic contrast injection, difficult cannulation, precut sphincterotomy, and papillary balloon dilation in an intact papilla [13]. Studies have shown that higher endoscopist and center procedural volumes are associated with greater technical success and lower overall adverse event rates, although no statistically significant volume-related differences have been observed for pancreatitis, cholangitis, or perforation when assessed individually [14]. Risk assessment before and during ERCP should therefore integrate the individual patient profile, stone burden and anatomical complexity, cannulation difficulty, and the experience and volume of the treating service, rather than attributing all complications to a single determinant.

Complication prevention should be embedded in procedural care rather than considered only after an event occurs. ESGE recommends routine rectal administration of 100 mg diclofenac or indomethacin immediately before ERCP in patients without contraindications to nonsteroidal anti-inflammatory drugs. In selected patients at high risk of PEP, prophylactic pancreatic stenting is recommended, including after inadvertent pancreatic duct guidewire insertion or opacification and double-guidewire cannulation [15]. Antibiotic prophylaxis is not recommended routinely, but should be considered when incomplete biliary drainage is anticipated, in severely immunocompromised patients, and during cholangioscopy [15]. Examining the delivery of these measures and the adverse events observed shortly after the procedure can identify practical opportunities for quality improvement.

Acute cholangitis is a potentially severe infectious complication of biliary obstruction in which prompt antimicrobial treatment and endoscopic source control are central to management. European guidance recommends endoscopic biliary drainage with urgency determined by disease severity. Recent ERCP-based bile-culture cohorts show that the microbiology is often polymicrobial and includes both enteric Gram-negative bacilli and enterococci. In a 2024 retrospective cohort of 880 patients with choledocholithiasis and biliary tract infection, 90.3% of ERCP-obtained bile cultures were positive and 37.5% were polymicrobial; *Escherichia coli* (30.4%) and *Klebsiella pneumoniae* (14%) predominated among Gram-negative isolates, whereas *Enterococcus faecium* (14%) was the leading Gram-positive organism [16]. Because organism distribution and resistance patterns vary between settings, bile cultures obtained during ERCP may be particularly useful for adapting antimicrobial therapy to local epidemiology and individual susceptibility results [16,17].

Local evidence is particularly valuable because tertiary-center cohorts may differ materially from the populations represented in multicenter studies. A tertiary-center cohort of patients with concomitant gallbladder and CBD stones illustrates that management strategy, procedure duration, length of stay, and pancreatitis rates may vary across organizational approaches [18]. They may include higher proportions of older adults, transferred patients with sepsis or pancreatitis, difficult stones, altered anatomy, and procedures requiring advanced techniques [10]. A retrospective cohort study can characterize these patients in real-world practice, describe ERCP management, and estimate the frequency and pattern of early adverse events. Relating clinical and procedural characteristics to early outcomes may also generate clinically useful hypotheses for patient selection, prophylaxis, and post-procedure monitoring.

Accordingly, this study aims to describe the clinical characteristics of patients with choledocholithiasis managed at a Romanian tertiary referral center, to evaluate ERCP procedural techniques, and to assess early adverse events. These findings can provide a useful reference for clinical practice, facilitate comparisons with other centers, and help identify areas where management could be further optimized. Most published ERCP cohorts focus on selected clinical scenarios such as post-ERCP pancreatitis, difficult cannulation, or acute cholangitis. Comprehensive real-world data describing the entire diagnostic work-up, endoscopic management, and early clinical outcomes of consecutive patients with choledocholithiasis treated in high-volume tertiary centers remain limited. Also, limited data exist regarding the epidemiological characteristics and clinical approach to choledocholithiasis in Eastern European populations, particularly in Romania, so the study addresses this evidence gap and aims to highlight the real clinical experience and insights into the management of choledocholithiasis in a high-volume tertiary referral center.

## 2. Materials and Methods

### 2.1. Study Design and Patient Selection

The study was designed as a retrospective, observational, cohort study and was conducted at Colentina Clinical Hospital, a tertiary referral center in Bucharest, Romania. The study was approved by the institution’s ethics committee and was conducted in accordance with the principles of the Declaration of Helsinki and data protection regulations including the General Data Protection Regulation (GDPR) 2016/679. Data were retrospectively collected and analyzed following approval by the institutional ethics committee.

During the study period (January 2021 to September 2025), 2514 ERCP procedures were performed at our tertiary referral center for all indications and recorded in a prospectively maintained endoscopy database. From this database, unique adult patients with confirmed choledocholithiasis were identified and assessed for eligibility. The individual patient was the unit of analysis, and each patient was included only once in the study. Complete-case analysis was performed, with exclusion of patients presenting missing values for any variable included in the study. No imputation procedures were applied for missing data. For patients who underwent more than one ERCP, only the first procedure was included in the analysis and was considered the index ERCP. All collected data were entered into a standardized database, and patient identifiers were removed to maintain confidentiality and protect privacy. The final cohort comprised 631 patients.

**Figure 1:**
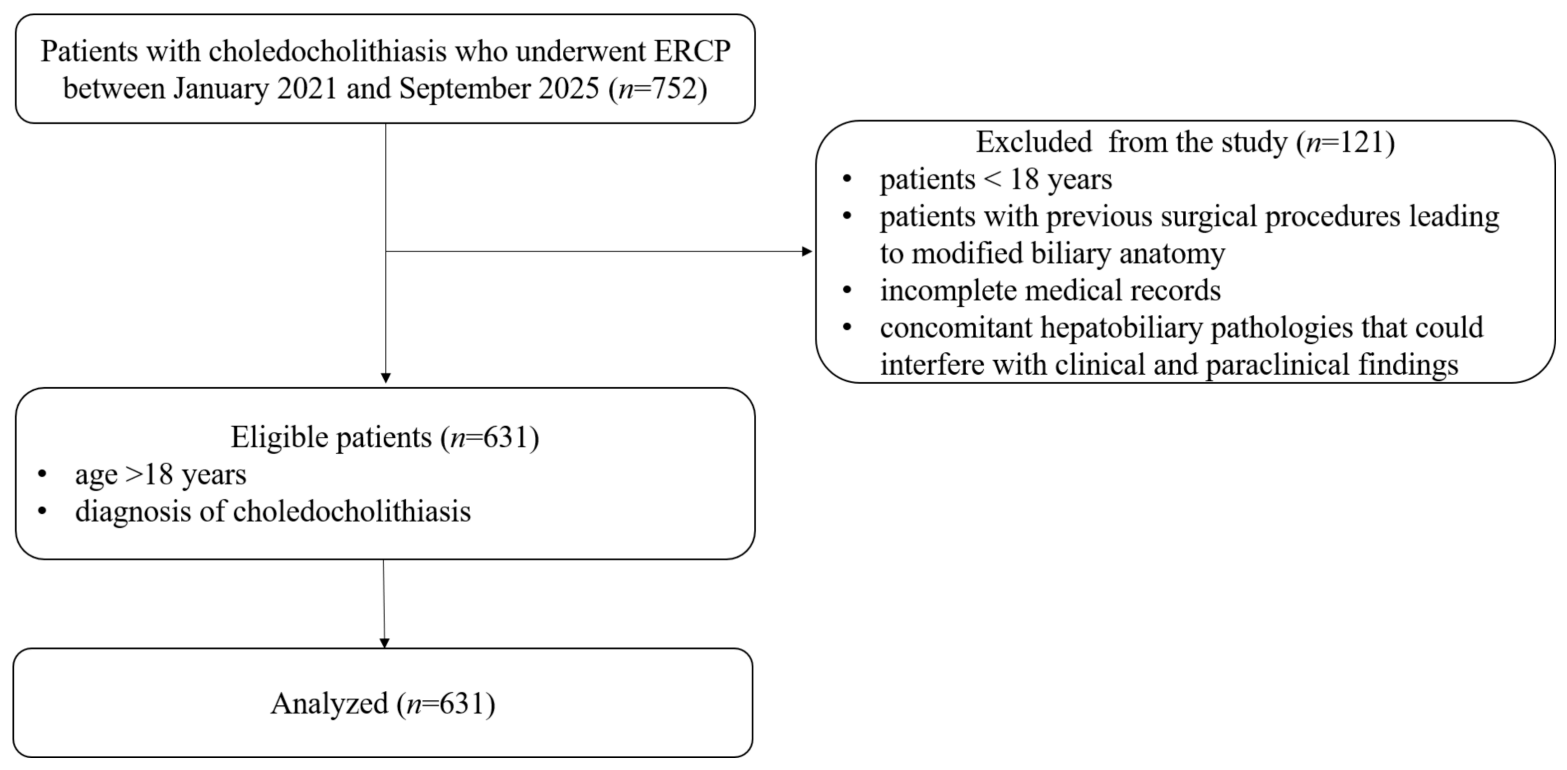
Flowchart showing patient selection process and the final study cohort.

Eligible participants were adults aged ≥18 years who underwent ERCP and had choledocholithiasis confirmed. Choledocholithiasis was diagnosed either at referring hospitals before transfer to our tertiary referral center or directly at our institution, based on clinical presentation, laboratory abnormalities (elevated liver enzymes and bilirubin), and imaging findings (transabdominal ultrasound, computed tomography (CT), magnetic resonance cholangiopancreatography (MRCP), or endoscopic ultrasound (EUS)). Exclusion criteria comprised incomplete medical records, concomitant hepatobiliary malignancies, other biliary pathologies requiring alternative therapeutic interventions, and previous major abdominal surgery resulting in altered anatomy. Following the application of these criteria, 631 patients were retained for final analysis. A flow diagram illustrating patient selection and exclusion is presented in Figure 1.

### 2.2. Data Acquisition and Study Variables

Data were extracted from the electronic hospital database, medical charts, discharge summaries, imaging records, and laboratory files. Patient identifiers were anonymized. We recorded age, sex, residence, medical history, prior cholecystectomy, medication exposure, smoking status, alcohol intake, symptoms, imaging, laboratory results, ERCP techniques, and clinical outcomes.

A range of laboratory parameters, including complete blood count, inflammatory markers: C-reactive protein (CRP), liver function tests (alanine aminotransferase (ALT), aspartate aminotransferase (AST), alkaline phosphatase (ALP), gamma-glutamyl transferase (GGT), total bilirubin (TB). All laboratory parameters analyzed in the study were measured exclusively at our center within 2 h of patient presentation, regardless of whether admission occurred through emergency care, scheduled hospitalization, or interhospital transfer.

A proportion of patients were referred from other centers with an established diagnosis of choledocholithiasis based on prior imaging studies. For study purposes, all available imaging examinations documented in the medical records, whether performed at our institution or at the referring centers, were considered in the analysis. Patients could undergo more than one imaging investigation during the diagnostic work-up; however, for the present analysis, the imaging modality that confirmed the diagnosis of choledocholithiasis was recorded for each patient, whether performed at our institution or at the referring center. Imaging findings from ultrasonography, CT, EUS, MRCP, and ERCP included details such as stone size and bile duct dilation. Details of the procedural techniques used were documented: cannulation success, sphincterotomy, stone extraction methods, stent placement and others.

Acute cholangitis present at admission was diagnosed and graded using Tokyo Guidelines 2018 (TG18) criteria [19]. ERCP-related adverse events were assessed separately. Immediate complications such as PEP, bleeding, perforation and other adverse events were noted. PEP was diagnosed following the Revised Atlanta Classification (2012) requiring at least two of the following three criteria: typical abdominal pain, serum amylase or lipase levels greater than three times the upper limit of normal, and characteristic imaging findings [20]. PEP severity was graded according to the Cotton criteria as mild, moderate, or severe, taking into account the duration of hospitalization and the occurrence of associated complications. Post-ERCP lipase measurement was not routinely performed in all patients; it was typically obtained 3–6 h after the procedure in patients who developed abdominal pain requiring symptomatic analgesic treatment. As part of the standard PEP prevention protocol, rectal indomethacin was administered to all patients undergoing ERCP. This was complemented by intravenous Ringer’s solution after the procedure, initiated with a 20 mL/kg bolus and continued thereafter as an infusion, in line with guideline recommendations. All patients, including those referred from other hospitals, were admitted and monitored in our institution following ERCP, ensuring standardized post-procedural surveillance and outcome assessment. All patients underwent clinical assessment for peritoneal signs at 24 and 48 h post-procedure; CT imaging was obtained for any patient with fever, abdominal pain, or leukocytosis. All patients received standard prophylaxis against PEP consisting of rectal diclofenac (100 mg) administered immediately before or after the procedure, together with routine post-procedural intravenous hydration according to the institutional protocol. Bleeding events occurring during ERCP were recorded prospectively and classified according to the American Society of Gastrointestinal Endoscopy (ASGE) lexicon [21], with severity determined by the clinical consequences of the event, including prolongation of hospitalization and the need for additional interventions. Difficult biliary cannulation was defined as either prolonged cannulation time (five minutes of active attempts at biliary cannulation), repeated attempts (more than five separate cannulation attempts) or repeated pancreatic duct instrumentation (at least two passages of a guidewire into the pancreatic duct in an effort to achieve biliary cannulation).

### 2.3. Statistical Analysis

Statistical analyses were performed using SPSS version 26.0 (IBM Corp., Armonk, NY, USA). Data collected were analyzed using descriptive statistics. Categorical variables were summarized as frequencies and percentages. Continuous variables were summarized as mean ± standard deviation (SD) or median (interquartile range [IQR]), as appropriate according to their distribution. 95% confidence intervals (CIs) were calculated using the Clopper–Pearson method. Results are presented in tables and figures for ease of interpretation. No inferential statistical tests were performed, as the study’s primary objective was to describe the patterns and prevalence of the variables of interest.

## 3. Results

During the study period, 2514 ERCP procedures were performed for all indications. Among patients evaluated for choledocholithiasis, 752 met the initial case definition and 631 unique patients were included in the study. Of these, 378 (59.9%) were female and 253 (40.1%) were male. Most participants resided in rural areas (*n* = 470, 74.5%), while 161 (25.5%) lived in urban areas. Age ranged from 20 to 99 years, with a median age of 68 years (IQR 56–77). Current smoking was reported by 200 patients (31.7%), and chronic alcohol use was reported by 489 patients (77.5%). Cardiovascular comorbidities were present in 454 patients (71.9%). Obesity, defined as a body mass index (BMI) greater than 30 kg/m^2^, was documented in 115 patients (18.2%), while type 2 diabetes mellitus was present in 130 patients (20.6%). Previous cholecystectomy was reported in 230 patients (36.5%). The study data regarding the general characteristics of the population are detailed in Table 1.

At presentation, the most frequently reported symptom was abdominal pain, observed in 374 patients (59.3%). Jaundice was present in 304 patients (48.2%). Nausea and/or vomiting were reported by 170 patients (26.9%), while fever, defined as a body temperature greater than 37.5 °C, was documented in 80 patients (12.7%).

Acute cholangitis was diagnosed at admission in 364 patients (57.7% of the overall cohort, 95%CI: 53.7–61.6%). Among the 364 patients with acute cholangitis, 192 (52.7%) were classified as mild, 39 (10.7%) as moderate, and 133 (36.6%) as severe according to the TG18 severity classification.

Diagnostic evaluation was based on cross-sectional and ultrasonographic imaging. The imaging modality confirming the diagnosis of choledocholithiasis was CT in 268 patients (42.5%), MRCP in 194 (30.7%), and abdominal ultrasonography in 169 (26.8%).

From a laboratory perspective, inflammatory markers were only mildly elevated, with a median CRP of 10.22 mg/L (IQR: 3.02–42.3) and a median white blood cell count of 7.53 × 10^3^/µL (IQR: 6.07–9.32). Liver biochemistry demonstrated a predominantly cholestatic pattern, with elevated median ALP (211.5 U/L, IQR: 110–379) and GGT (358 U/L, IQR: 126–718), accompanied by moderate elevations in transaminases and bilirubin. The laboratory profile of the study cohort at the time of hospital admission is summarized in Table 2.

Anatomical characteristics, stone-related features, and ERCP-related procedural variables were analyzed to characterize the endoscopic management of the study cohort. The median CBD diameter was 14 mm (IQR: 10–17), while the median stone diameter was 7.9 mm (IQR: 3–12). Multiple stones or biliary sludge were identified in 358 patients (56.7%), and a duodenal diverticulum was present in 111 (17.6%). Difficult biliary cannulation occurred in 68 patients (10.8%), with precut sphincterotomy required in 64 (10.1%). Unintentional MPD cannulation and contrast injection occurred in 96 (15.2%) and 30 (4.8%) patients, respectively, while prophylactic pancreatic stent placement was performed in 33 cases (5.2%). Balloon-assisted stone extraction attempts were performed in all patients (100%), whereas mechanical lithotripsy and dilation balloon assistance were required in 123 (19.5%) and 16 (2.5%) patients, respectively. Biliary stents were placed in 119 patients (18.9%), spontaneous stone passage was documented in 79 (12.5%), and bile cultures were obtained in 52 patients (8.3%). In-hospital mortality occurred in 2 patients (0.3%). All the ERCP procedural techniques and related complications are detailed in Table 3.

Post-ERCP complications were assessed, with PEP being the most common adverse event. PEP developed in 68 patients (10.8%), with a median post-ERCP serum lipase level of 1391 U/L (IQR: 414–5270.5). Intraprocedural bleeding occurred in 31 cases (4.9%), all of which were mild and were successfully managed with on-site endoscopic hemostasis (epinephrine injection, balloon compression, electrocoagulation, or hemostatic clip placement). Post-ERCP bleeding occurred in 12 patients (1.9%). No cases of perforation were documented. The incidence of post-ERCP complications is summarized in Table 4.

Bile cultures were performed in 52 patients (8.3%). Of these, 15 cultures (2.4% of the overall cohort) showed no bacterial growth. Among positive cultures, Gram-negative enteric organisms predominated, with *Escherichia coli* representing the most common isolate (11 patients, 1.7%), followed by *Klebsiella oxytoca*. Enterococcal species were also frequently identified, including high-level aminoglycoside-resistant (HLAR) *Enterococcus faecium* isolates. Less common findings included *Enterobacter, Klebsiella, Pseudomonas*, and several polymicrobial infections. HLAR phenotype was identified in 3 *Enterococcus faecium* isolates, and ESBL production was detected in 1 *Klebsiella pneumoniae* isolate. Details about the microbiological findings are documented in Table 5.

## 4. Discussion

This retrospective tertiary-center cohort describes a clinically consequential burden of choledocholithiasis: patients were older, predominantly female, frequently multimorbid, and more than half fulfilled criteria for acute cholangitis on admission. Interpretation should account for the referral setting. A center that performs therapeutic ERCP is likely to receive patients with persistent obstruction, cholangitis, complex stone burden, or unsuccessful initial management. The cohort therefore should not be read as a population-based estimate of uncomplicated CBD stones. The female predominance, older age and co-occurrence of diabetes are consistent with recent literature. A contemporary tertiary-center study identified female sex, middle age and diabetes among the reported risk factors, although comparisons with small single-center studies should be made cautiously because their case definitions and referral pathways differ [22]. In the present cohort, the high prevalence of cardiovascular comorbidity, diabetes, prior cholecystectomy, and rural residence further suggests a population in whom comorbidity, access to early assessment, and delayed referral may have shaped the threshold for admission and ERCP.

The symptom profile is clinically coherent with intermittent or persistent biliary obstruction: abdominal pain and jaundice were common, whereas fever was documented in only a minority. This divergence from the classical Charcot triad should not be interpreted as evidence against cholangitis. One study found that the diagnostic accuracy of Charcot’s triad was only 67.8%, compared with 92.3% for the Tokyo diagnostic framework [23]. The high proportion classified as cholangitis in our series therefore more likely reflects the broader, objective TG18 framework, which integrates systemic inflammation, cholestasis and imaging, than a paradoxically mild clinical syndrome. The substantial proportion of severe cases is notable. In an Australian study, in-hospital mortality was 5% in grade III versus 1.4% in grade I disease [24]. A tertiary referral population with a higher proportion of patients presenting with organ dysfunction and transferred cases could plausibly explain why severe cholangitis was more frequent here than in a Romanian single-center cholangitis cohort (15%) [25]. Conversely, the relatively small moderate-grade group may reflect how patients fulfilled the TG18 severity criteria in this cohort, with many classified as either mild or severe, rather than indicating a true biological discontinuity between severity grades.

The admission biochemical pattern supports mechanical biliary obstruction as the dominant process, with caution, despite more than half of the cohort meeting the diagnostic criteria for acute cholangitis and the upper quartile for CRP being only 42.3 mg/L; as pooled laboratory values include patients with and without infection, these figures may have been obtained before peak inflammatory response or after antibiotic initiation, and may be attenuated in older or multimorbid adults. TG18 does not require all inflammatory markers to be markedly abnormal. Its value lies in integrating inflammation, cholestasis and imaging, and has shown high diagnostic accuracy in independent cohorts [19]. Thus, the laboratory findings are compatible with cholestatic obstruction but should not be used alone to reduce the level of clinical concern in jaundiced patients with imaging evidence of biliary obstruction.

In our cohort, CT was more frequently documented than MRCP and abdominal ultrasonography, likely reflecting the imaging strategy routinely applied in our department, together with imaging studies performed before referral in transferred patients, rather than adherence to an optimal imaging algorithm. This approach differs from the diagnostic sequence recommended by ESGE, which places liver tests plus abdominal ultrasonography first and reserves MRCP or EUS for persistent suspicion after inconclusive ultrasonography [6]. CT can demonstrate ductal dilatation, complications and competing diagnoses but may miss small or non-calcified stones; MRCP enables non-invasive visualization of the biliary tree and is particularly helpful when the diagnosis remains uncertain after initial clinical and laboratory assessment. In this cohort, a median 14 mm duct and a median 7.9 mm stone make clinically significant obstruction plausible, while the 12.5% rate of documented spontaneous passage also illustrates why diagnostic confirmation immediately before an invasive procedure matters. The imaging mix should therefore be discussed as a marker of acute-care case selection and local access, rather than as proof that CT is the preferred test for all suspected stones.

Endoscopic findings indicate a mixed population of standard and complex stone disease. Multiple stones or biliary sludge, bile duct dilatation, periampullary diverticula, and larger stone size can all complicate stone extraction by reducing the effective distal duct-to-stone ratio or making papillary orientation more challenging. Contemporary reviews estimate that approximately 10–15% of stone-extraction ERCPs are complex or difficult [26]. In this context, the proportions of patients undergoing mechanical lithotripsy (19.5%) and biliary stenting (18.9%) are compatible with referral enrichment for stones that were multiple, large relative to the distal duct, impacted, or not safely cleared in one session. Temporary plastic stenting is guideline-concordant when stones are not retrievable but drainage is required [6]. Current ESGE guidance favors limited sphincterotomy plus endoscopic papillary large-balloon dilation as first-line treatment for difficult stones, with cholangioscopy-guided electrohydraulic or laser lithotripsy as an effective escalation strategy. The limited use of dilation-assisted extraction likely reflects institutional practice, local case mix, operator preference, and the procedural strategy adopted during the study period, including earlier use of mechanical lithotripsy. Consequently, it should not be interpreted as indicating inferior performance in the absence of data on stone clearance at the index ERCP, stone impaction, distal bile duct caliber, and repeat interventions.

Difficult cannulation and precut sphincterotomy were recorded in 10.8% and 10.1% of patients, respectively, and should be interpreted together, rather than as isolated operator-performance measures. ESGE defines difficult cannulation by more than 5 min or 5 papillary contacts, or more than one unintended pancreatic-duct access/opacification [27]. Periampullary diverticula, altered papillary anatomy, inflammation, impacted stones and repeated pancreatic access can all move a case into this category. The close correspondence between difficult cannulation and precut use likely reflects the procedural strategy routinely adopted in our department, with early transition to a rescue access technique after failed standard cannulation. However, MPD cannulation occurred in 96 patients (15.2%) and contrast opacification in 4.8%, both recognized procedure-related risk factors for PEP, while prophylactic pancreatic stents were placed in 33 (5.2%). Because the specific indications for prophylactic pancreatic stent placement were not consistently documented in this retrospective cohort, the appropriateness of stent use relative to pancreatic duct instrumentation could not be assessed. Also, the proportion of patients receiving prophylactic pancreatic stent placement (5.2%) should not be interpreted in relation to the entire cohort but rather to the subgroup of patients who underwent MPD manipulation. The ESGE recommends guidewire-assisted biliary cannulation as the primary technique, pancreatic guidewire-assisted cannulation when repeated unintended pancreatic duct access occurs, and prophylactic pancreatic stent placement in patients undergoing pancreatic guidewire-assisted cannulation or other selected high-risk cannulation techniques [27]. Because all patients received rectal diclofenac and institutional intravenous hydration, the observed PEP rate cannot be attributed simply to absence of standard pharmacological prophylaxis; it more likely reflects residual patient- and procedure-level risk, particularly in patients with repeated pancreatic-duct instrumentation or complex access.

Bile microbiology was directionally consistent with ascending infection from enteric flora: *E. coli* predominated, followed by *Klebsiella* species, with *Enterococcus* spp., including HLAR *E. faecium*, also recovered. This is concordant with a 2024 cohort of 1063 cholangitis patients in which *E. coli*, *Klebsiella, Enterococcus* and *Pseudomonas* were the four most common organisms in blood and bile [28]. It is also consistent with a large ERCP bile-aspirate series, in which *Enterococcus, Klebsiella* and *E. coli* were frequent and 7.9% of *Enterobacteriaceae* were extended-spectrum β-lactamase (ESBL) producers [17]. The recovery of resistant enterococci is clinically important because community-acquired biliary infection is not microbiologically uniform and prior interventions, antibiotics, stents or sphincterotomy can select for enterococci and more resistant organisms. Recent prospective data from Bucharest and Bordeaux underscore that local microbiological patterns matter: *E. coli* was most common in the Bucharest arm, while multidrug-resistant isolates represented 21.2% of strains there versus 7.2% in Bordeaux [29]. In our cohort, bile cultures were selectively obtained in patients with clinical suspicion of biliary infection or according to operator preference, rather than as part of a standardized study protocol. Because cultures were obtained in only 8.3% of this cohort, the observed isolate distribution is susceptible to indication bias toward more severe, recurrent or treatment-refractory cases and should not be extrapolated to all patients with choledocholithiasis. Nevertheless, these findings support the potential clinical utility of bile culture sampling in selected patients with acute cholangitis, prior biliary instrumentation, severe illness, or an inadequate clinical response, where the results may guide antimicrobial de-escalation or escalation. ESGE specifically recommends bile sampling during repeat ERCP for post-ERCP cholangitis that does not improve conservatively [15].

PEP was the most common adverse event, occurring in 10.8% of patients. This rate is comparable to the mean incidence of 12.2% reported in a recent meta-analysis of 55 randomized trials, although the included studies were heterogeneous and often involved patients at increased procedural risk rather than unselected cohorts with choledocholithiasis [30]. It is higher than the 5.3% pancreatitis rate reported in a 283-patient Romanian cholangitis ERCP cohort, in which bleeding was the leading complication [25]. The difference is biologically and procedurally plausible: recurrent MPD access, contrast injection, difficult cannulation and precut sphincterotomy cluster in the present series and are mechanisms for papillary and pancreatic injury. The uniform administration of rectal diclofenac and routine intravenous hydration, together with inpatient observation of every patient and protocolized clinical assessment at 24 and 48 h, makes under-ascertainment of early clinically apparent adverse events less likely than in datasets relying on outpatient follow-up or referring-hospital records. Difficult cannulation, repeated pancreatic duct instrumentation, and precut sphincterotomy are established procedure-related risk factors for PEP and are therefore relevant when interpreting the observed complication rate in our cohort. The 4.9% intraprocedural bleeding rate, all successfully controlled endoscopically and graded mild without subsequent clinical consequences, is reassuring with respect to immediate hemostatic capability but should not be directly equated with delayed clinically significant post-sphincterotomy bleeding. The absence of perforation is compatible with its rarity but, in a retrospective study of this size, does not establish absence of risk. In-hospital mortality in our cohort was 0.3%. This rate is comparable to the low mortality reported after ERCP in the literature. A recent large systematic review and meta-analysis including 380 studies reported an ERCP-attributable mortality of 0.2% (95% CI 0.1–0.3%), while higher mortality rates have been described in older and higher-risk populations [12]. However, direct comparisons should be interpreted cautiously, as our study recorded all-cause in-hospital mortality rather than mortality specifically attributable to ERCP.

Taken together, the present cohort provides a comprehensive snapshot of contemporary ERCP practice for choledocholithiasis, encompassing patient presentation, diagnostic evaluation, procedural characteristics, microbiological findings, and early clinical outcomes within a high-volume tertiary referral center. These findings provide a descriptive overview of the ERCP care pathway in our center and may help identify areas for local clinical audit and quality improvement, including diagnostic imaging, biliary drainage, PEP prophylaxis, rescue cannulation techniques, and targeted bile culture sampling when clinically indicated.

### Study Limitations

Several limitations should be acknowledged when interpreting these results. First, the retrospective, observational design limits causal inferences and may introduce selection bias. Second, in a tertiary referral center, our cohort likely includes more complex cases than those typically encountered in general practice so the study is likely enriched for cholangitis, comorbidity, challenging stone disease and complex ERCP, limiting direct generalizability to unselected patients with choledocholithiasis or to primary-care settings. Third, comparisons between clinical subgroups may be confounded by referral timing, prior treatment at outside hospitals, stone burden, anatomical factors and operator-level decision-making. Given the descriptive design and absence of inferential or adjusted analyses, the present study cannot identify independent risk factors or predictors of clinical outcomes. Limited long-term follow-up restricts the assessment of late complications. Another limitation is that information on antibiotic therapy administered before transfer was not consistently available for patients referred from other hospitals. Consequently, inflammatory markers and microbiological findings at admission may have been influenced by prior treatment, potentially leading to an underestimation of the inflammatory response or reduced bile culture yield. Several relevant procedural and follow-up variables were not systematically recorded, including the timing of ERCP and biliary drainage, complete duct clearance, the number of ERCP sessions, indications for temporary biliary stenting, and long-term endoscopic follow-up. Consequently, the overall therapeutic success rate of ERCP could not be reliably determined, and the potential impact of time to biliary drainage on clinical outcomes could not be assessed. Finally, bile cultures were obtained in a small, clinically selected subset, so the microbiological profile and resistance findings cannot be regarded as prevalence estimates for the full cohort.

## 5. Conclusions

This descriptive study provides a comprehensive real-world characterization of patients with choledocholithiasis undergoing ERCP in a high-volume tertiary referral center. The findings reflect a clinically complex population, with acute cholangitis present in 57.7% of patients and frequent need for additional therapeutic interventions, including mechanical lithotripsy in 19.5% and biliary stent placement in 18.9%. Despite this complexity, major early adverse outcomes were uncommon, with post-ERCP bleeding occurring in 1.9% and in-hospital mortality in 0.3%, although PEP remained the most frequent adverse event (10.8%). Taken together, the clinical, diagnostic, procedural, microbiological, and outcome data provide a contemporary picture of ERCP management for choledocholithiasis in tertiary care. From a practical perspective, these real-world findings contribute to the understanding of how choledocholithiasis is managed in routine clinical practice and support a multidisciplinary approach. The cohort may also provide a useful real-world reference for comparison with other tertiary centers and healthcare settings, while differences in referral patterns and case complexity should be considered when interpreting such comparisons. Future research should focus on prospective multicenter studies, long-term follow-up, and standardized care protocols to further improve the quality of management for this condition.

## Figures and Tables

**Table 1:** General characteristics of the study population.

Variable	Value
Total patients, *n* (%)	631 (100%)
Female, *n* (%)	378 (59.9%)
Male, *n* (%)	253 (40.1%)
Urban residence, *n* (%)	161 (25.5%)
Rural residence, *n* (%)	470 (74.5%)
Age range, years	20–99
Median age [IQR], years	68 [56–77]
Current smokers, *n* (%)	200 (31.7%)
Chronic alcohol use, *n* (%)	489 (77.5%)
Cardiovascular comorbidities, *n* (%)	454 (71.9%)
Obesity (BMI > 30 kg/m^2^), *n* (%)	115 (18.2%)
Type 2 diabetes mellitus, *n* (%)	130 (20.6%)
Previous cholecystectomy, *n* (%)	230 (36.5%)

**Table 2:** Laboratory Parameters at Admission.

Parameter	Median [IQR]
CRP (mg/L)	10.22 [3.02–42.3]
WBC count (×10^3^/µL)	7.53 [6.07–9.32]
Neutrophil count (×10^3^/µL)	4.85 [3.64–6.64]
Lymphocyte count (×10^3^/µL)	1.63 [1.23–2.19]
TB (mg/dL)	1.13 [0.56–2.83]
AST (U/L)	41.9 [21.4–93.3]
ALT (U/L)	65.5 [25.0–157.4]
ALP (U/L)	211.5 [110–379]
GGT (U/L)	358 [126–718]

**Table 3:** ERCP-related procedural characteristics and techniques.

Variable	Value	95% CI
CBD diameter, median [IQR], mm	14 [10–17]	-
Stone diameter, median [IQR], mm	7.9 [3–12]	-
Multiple stones/sludge, *n* (%)	358 (56.7%)	52.9–60.7
Duodenal diverticulum present, *n* (%)	111 (17.6%)	14.7–20.8
Difficult biliary cannulation, *n* (%)	68 (10.8%)	8.5–13.5
Precut sphincterotomy, *n* (%)	64 (10.1%)	7.9–12.8
MPD cannulation, *n* (%)	96 (15.2%)	12.5–18.3
MPD contrast injection, *n* (%)	30 (4.8%)	3.2–6.7
Prophylactic pancreatic stent placement, *n* (%)	33 (5.2%)	3.6–7.3
Balloon-assisted stone extraction attempted, *n* (%)	631 (100%)	99.4–100
Mechanical lithotripsy, *n* (%)	123 (19.5%)	16.5–22.8
Dilation balloon use, *n* (%)	16 (2.5%)	1.5–4.1
Biliary stent placement, *n* (%)	119 (18.9%)	15.9–22.1
Spontaneous stone passage, *n* (%)	79 (12.5%)	10–15.4
Intraprocedural bleeding, *n* (%)	31 (4.9%)	3.4–6.9
Bile culture obtained, *n* (%)	52 (8.3%)	-

**Table 4:** Frequency and severity of post-ERCP complications.

Variable	Value	95% CI
PEP, *n* (%)	68 (10.8%)	8.5–13.5
Post-ERCP serum lipase, median [IQR], U/L	1391 [414–5270.5]	-
PEP severity, *n* (%)		
Mild	48 (70.7%)	
Moderate	15 (22.0%)	
Severe	5 (7.3%)	
Post-ERCP bleeding, *n* (%)	12 (1.9%)	1–3.3
In-hospital mortality, *n* (%)	2 (0.3%)	0–1.1

**Table 5:** Microbiological Findings in Bile Cultures.

Bile Culture Result	*n* (% *)
Sterile (no growth)	15 (28.8%)
*Escherichia coli*	11 (21.1%)
*Klebsiella oxytoca*	4 (7.7%)
*Enterococcus faecium*	6 (11.5%)
*Klebsiella pneumoniae*	3 (5.8%)
*Enterobacter cloacae* complex	2 (3.8%)
*Enterococcus faecalis*	2 (3.8%)
*Enterococcus* spp.	2 (3.8%)
*Escherichia coli* + *Enterococcus faecium*	1 (1.9%)
*Escherichia coli* + *Proteus* spp.	1 (1.9%)
*Escherichia coli* + *Enterococcus casseliflavus*	1 (1.9%)
*Enterobacter cloacae*	1 (1.9%)
*Klebsiella* spp. + *Pseudomonas* spp.	1 (1.9%)
*Pseudomonas aeruginosa* + *Enterococcus* spp.	1 (1.9%)
*Pseudomonas* spp.	1 (1.9%)

* Percentages are calculated using the 52 patients who underwent bile culture as the denominator.

## Data Availability

The datasets generated during and/or analyzed during the current study are available from the corresponding author under reasonable request.

## Abbreviations

The following abbreviations are used in this manuscript:

AC	acute cholangitis
ALP	alkaline phosphatase
ALT	alanine aminotransferase
ASGE	American Society of Gastrointestinal Endoscopy
AST	aspartate aminotransferase
BMI	Body Mass Index
CBD	common bile duct
CRP	C-reactive protein
CT	computed tomography
ERCP	endoscopic retrograde cholangiopancreatography
ESBL	Extended-Spectrum β-Lactamase
ESGE	European Society of Gastrointestinal Endoscopy
EUS	endoscopic ultrasound
GGT	gamma-glutamyl transferase
HLAR	High-Level Aminoglycoside Resistance
IQR	interquartile range
MPD	main pancreatic duct
MRCP	magnetic resonance cholangiopancreatography
PEP	post-ERCP acute pancreatitis
SD	standard deviation
TB	total bilirubin
TG18	Tokyo Guidelines 2018
WBC	white blood cells
